# Integrated Analysis of DNA methylation and transcriptome profile to identify key features of age-related macular degeneration

**DOI:** 10.1080/21655979.2021.1976502

**Published:** 2021-09-27

**Authors:** Zhijie Wang, Yinhua Huang, Feixue Chu, Kai Liao, Zekai Cui, Jiansu Chen, Shibo Tang

**Affiliations:** a Aier School of Ophthalmology, Central South University, Changsha, China; b Aier Eye Institute, Changsha, China; c Hangzhou Xihu Zhijiang Eye Hospital, Hangzhou, China; d Key Laboratory for Regenerative Medicine, Ministry of Education, Jinan University, Guangzhou, China; e Institute of Ophthalmology, Medical College, Jinan University, Guangzhou, China; f Cas Center for Excellence in Brain Science and Intelligence Technology, Chinese Academy of Sciences, Shanghai, China

**Keywords:** Age-related macular degeneration disease, DNA methylation, transcriptome profile, immune cell infiltration, data integration, key genes

## Abstract

Age-related macular degeneration (AMD) is a common vision-threatening disease. The current study sought to integrate DNA methylation with transcriptome profile to explore key features in AMD. Gene expression data were obtained from the Gene Expression Omnibus (GEO, accession ID: GSE135092) and DNA methylation data were obtained from the ArrayExpress repository (E-MTAB-7183). A total of 456 differentially expressed genes (DEGs) and 4827 intragenic differentially methylated CpGs (DMCs) were identified between AMD and controls. DEGs and DMCs were intersected and 19 epigenetically induced (EI) genes and 15 epigenetically suppressed (ES) genes were identified. Immune cell infiltration analysis was performed to estimate the abundance of different types of immune cell in each sample. Enrichment scores of inflammatory response and tumor necrosis factor-alpha (TNFα) signaling via nuclear factor kappa B (NF-κb) were positively correlated with abundance of activated memory CD4 T cells and M1 macrophages. Subsequently, two significant random forest classifiers were constructed based on DNA methylation and transcriptome data. SMAD2 and NGFR were selected as key genes through functional epigenetic modules (FEM) analysis. Expression level of SMAD2, NGFR and their integrating proteins was validated in hydrogen peroxide (H_2_O_2_) and TNFα co-treated retinal pigment epithelium (RPE) *in vitro*. The findings of the current study showed that local inflammation and systemic inflammatory host response play key roles in pathogenesis of AMD. SMAD2 and NGFR provide new insight in understanding the molecular mechanism and are potential therapeutic targets for development of AMD therapy.

## Introduction

Age-related macular degeneration (AMD) is the most common vision-threatening disease in elderly people [1,2]. It is characterized by pigmentation abnormalities in retinal pigment epithelium (RPE), yellowish drusen and loss of photoreceptor in the macular region. In an advanced stage of AMD, invasion of choroidal neovascularization or geographic atrophy causes severe visual impairment, thus the individual cannot undertake basic activity and reduces the quality of life [2]. Studies predicted that approximately 288 million people will be affected by AMD globally by 2040 [3]. A high prevalence of AMD leads to a major burden on public health.

AMD is a multifactorial disorder implying that both environmental factors and genetic risks are involved in the onset and progression of AMD [2]. Environmental factors, such as aging, smoking and diet, play key roles in AMD. However, the mechanism of these factors has not been fully elucidated [4]. Epigenetic mechanisms are impressionable by external stimuli and mediate gene-environment interaction. Advances in high-throughput strategies, such as next-generation sequencing, have enables elucidation of numerous genetic and epigenetic pathogenesis factors. A genome-wide association study for AMD reported 34 genetic variants loci implicated in pathogenesis of AMD [5]. Several studies report that epigenetic abnormalities play a role in promoting AMD [6]. DNA methylation is a major epigenetic modification that is occurs by adding a methyl group to DNA molecules. Previous studies report aberrant DNA methylation in AMD, such as hypomethylation of IL17RC and differential methylation of SKI, GTF2H4 and TNXB [7–9]. Therefore, AMD-specific methylation markers and their detailed functions are potentially significant in investigating pathogenesis and treatment of AMD.

DNA methylation plays an important role in disease prediction and prognosis prediction, including carcinoma and Parkinson’s disease [10–13]. In addition, combining transcriptome and DNA methylation can help in identifying important genes implicated in the mechanism of the diseases and can serve as therapeutic targets [14,15]. However, only a few studies have integrated DNA methylation and transcriptome profile to explore key features of AMD. Therefore, the current study systematically explored DNA methylation level and gene expression level of AMD by analyzing RPE-sourced DNA methylation array and RNA-seq data. Furthermore, random forest classifiers were constructed based on methylation data and gene expression data to distinguish AMD from controls. In addition, functional epigenetic modules analysis was performed to explore key genes implicated in AMD.

## Methods

### Ethics statement and culture of human RPE cells

RPE tissue was provided by Aier Eye Bank (Changsha, China) and written informed consent was obtained from the donor. Ethical approval was obtained from the medical ethics committee of Aier Eye Hospital Group (AlER2018lRB21). All experimental procedures were in accordance with the Declaration of Helsinki. Isolation and culture of RPE cells was performed as described in our previous study [16]. The retina was removed from the ocular tissue under the microscope and the RPE layer was digested using 0.25% ethylenediaminetetraacetic acid (EDTA)-trypsinase (Gibco, USA) in the remainder of the eyecup. After centrifugation (100 g, 5 min), RPE cells were resuspended in culture media, which comprised DMEM/F12 (Gibco), 10% fetal bovine serum and 1% penicillin-streptomycin (Gibco). RPE cells were co-treated with 100 ng/ml tumor necrosis factor-alpha (TNFα) and 100 μM hydrogen peroxide (H_2_O_2_) for 24 h to simulate oxidative stress condition of AMD *in vitro*.

## Data collection

Gene expression data for RPE tissues from postmortem eyes were downloaded from Gene Expression Omnibus (GEO, accession ID: GSE135092) [17]. According to the original paper, postmortem eyes from 53 female and 76 male donors, ranging from 59 to 98 years of age, were procured by the Florida Lions Eye Bank (Tampa, FL). Eyes were enucleated 4 h postmortem or less to maintain the integrity of RNA. Grading of postmortem eyes was performed based on the Minnesota Grading System after removal of anterior segment and vitreous tissue [18]. Only eyes with category 1 disease (controls) and category 4 disease (advanced AMD) were used in the original study [17]. Bulk expression data of RPE tissues were extracted for the present study, comprising131 macular and 135 non-macular RPE samples. Macular samples were used for differential gene expression analysis, whereas non-macular samples were used to validate the accuracy of the random forest classifier. All data were generated with Illumina HiSeq2500 (Illumina, San Diego, CA, USA) and raw count values and RPKM (Reads Per Kilobase Million) values were obtained.

Genome-wide DNA methylation data containing RPE tissues of 19 normal controls and 25 AMD patients were downloaded from the ArrayExpress database (accession ID: E-MTAB-7183) (https://www.ebi.ac.uk/arrayexpress/experiments/E-MTAB-7183/) [7]. The postmortem eyes were obtained from the Manchester Eye Bank (UK) from donors aged over 50 years. Tissue phenotyping was also performed based on the Minnesota Grading System [19]. RPE samples from 19 normal control donors (level 1) and 25 AMD donors (level 2 and 3) were used for DNA methylation assay [7]. DNA methylation levels were determined using Illumina Infinium HumanMethylation450 (‘450 K array’) BeadChip (Illumina, San Diego, CA, USA) and raw IDAT files were obtained.

## Differential expression analysis

A total of 131 macular RPE-based samples were extracted for differential expression analysis between AMD samples and controls. The *edgeR* R package was used to determine differentially expressed genes (DEGs) based on raw read counts using generalized linear models [20]. DEGs were then defined based on a Benjamini-Hochberg adjusted *p*-value <0.05 and fold change >1.5.

## Differential methylation analysis

*ChAMP* R package was used to process DNA methylation data from the IDAT files generated by the Illumina HumanMethylaton450 platform [21]. Probes that met the following criteria were filtered: (1) detection p-value >0.01; (2) <3 beads in at least 5% of samples per probe; (3) non-CpG probes; (4) SNP-related probes; (5) multi-hit probes and (6) located in chromosome X and Y. After filtering, normalization was performed using the beta-mixture quantile normalization method and ComBat function in *sva* R package was used to adjust for the effect of array slides [22]. The normalized beta values were used to identify differentially methylated CpGs (DMCs) with a *p*-value <0.01. In addition to promoter methylation suppressing gene expression, DMCs can come exhibit other effects, such as gene body methylation-associated regulation [23]. Therefore, corresponding genes of intragenic DMCs were defined as differentially methylated genes (DMGs) regardless of the specific region.

## Functional enrichment analysis

Functional enrichment analysis was performed with the *clusterProfile* R package for the Kyoto Encyclopedia of Genes and Genomes (KEGG) pathways and Gene Ontology (GO) terms [24]. Significant terms were defined as terms with an adjusted *p*-value <0.05. Enrichment analysis results were visualized through *clusterProfile* and *GOplot* R package [24,25].

## Gene set enrichment analysis and Gene set variation analysis

For Gene set enrichment analysis (GSEA), the hallmark gene set of ‘h.all.v7.4.symbols.gmt’ was retrieved from MSigDB (https://www.gsea-msigdb.org/gsea/msigdb) and used as the reference. GSEA was performed using *clusterProfiler* R package and adjusted *p* < 0.05 indicated significance [24]. Gene set variation analysis (GSVA) is an unsupervised method that estimates variation of gene sets over a sample population [26]. In the present study, GSVA method was used to quantify significantly enriched gene sets identified in GSEA.

## Immune cell infiltration and correlation analysis

CIBERSORT is an analytical tool that provides an estimation of the abundance of 22 immune cell types in mixed cell tissue with bulk expression data [27]. CIBERSORT was used to explore infiltration of immune cells in RPE from the AMD cohort. Differences in immune cell composition between AMD and controls were determined using Wilcoxon sign rank test. Combination of GSVA and immune cell infiltration is useful in revealing the possible impact of immune infiltration in as described previously [28,29]. In the current study, correlation analysis between the proposition of immune cells and GSVA scores of enriched immune processes were performed using Spearman correlation analysis. A *p*-value <0.05 was considered statistically significant.

## Intersection of DEGs and DMGs

To explore the relationship between methylation level and gene expression, DMGs and DEGs were intersected to identify overlapping genes. Hypo-methylated genes with a high expression level referred as epigenetically induced (EI) and hypermethylated gene with a low expression level referred as epigenetically suppressed (ES) genes were extracted for subsequent analyses.

## Construction of random forest classifiers

The random forest algorithm is an ensemble machine learning method based on construction of several decision trees. In the current study, a random forest classifier was used to separately distinguish AMD patients from controls based on gene expression data and DNA methylation data. EI and ES genes were selected as candidate variables. A normalized beta value of CpGs was used to construct the classifier based on DNA methylation data. Log2 transformed count data was used to construct the classifier for gene expression. The Bioconductor package *randomForest* was used to run the random forest algorithm [30]. The importance of each variable was initially determined. Variables were added consecutively based on order of importance to identify the best classification capacity of classifiers with the leave-one-out cross-validation (LOOCV) method. The area under the curve (AUC) of the receiver operating characteristic (ROC) curve was then calculated to determine the quality of classifiers using *pROC* R package [31]. In addition, the non-macular RPE expression data in GSE135092 were used to validate the accuracy of the gene expression classifier.

## Functional epigenetic modules analysis

Functional epigenetic modules (FEM) analysis is a method for identification of interactome hotspots of differential methylation and differential expression [32]. FEM analysis was widely used to identify protein modules in integrative analysis of DNA methylation and transcriptome, such as Parkinson’s disease, colorectal cancer and Pituitary Adenomas [33–35]. FEM analysis was performed to further explore association between DNA methylation and gene expression under protein interaction networks. FEM analysis was performed using a Bioconductor package *FEM*, using RPKM values of gene expression data and normalized methylation beta values. The input parameters were set as follows: nseeds = 100, gamma = 0.5 and nMC = 1000. A significant module was defined by a *p*-value <0.05. Centric genes identified in FEM were selected for candidate key genes.

## Quantitative polymerase chain reaction (qPCR)

Total RNA was extracted from RPE cells in 12-well plates (n = 4 in each group) using Trizol reagent (Invitrogen, USA). Total RNA was reverse transcribed into cDNA using HiScript II Q Select RT SuperMix for qPCR (Vazyme, China). qPCR was performed using SYBR green reagent (Vazyme, China) on Roche 96 (Roche, USA). Gene expression level was quantified using 2^−ΔΔCt^ method. GAPDH (glyceraldehyde 3-phosphate dehydrogenase) was used as internal control gene. Analysis of each sample was performed in triplicate. Primer sequences are listed in Supplement Table S1. Statistical analysis was performed in Graphpad Prism software. Statistical difference between groups was assessed by Student’s t-test. p < 0.05 was considered statistically significant. Data were presented as mean ± SEM.

## Results

Integrated analysis of DNA methylation data and transcriptome profile provides information on the mechanism of diseases and provides a basis for exploring potential targets for further research. In the current study, DEGs, DMGs and enriched GO terms and KEGG pathways were identified in AMD. Further, immune infiltration analysis and GSVA analysis was conducted to explore the role of immune filtration in inflammation in AMD. Subsequently, DEGs and DEGs were intersected to identify epigenetically induced (EI) genes and epigenetically suppressed (ES) genes. Two classifiers were constructed using random forest algorithm to explore the prediction power of EI and ES genes in distinguishing AMD patients from controls. Finally, key genes were identified by FEM analysis and further validated in cultured RPE cell *in vitro*.

## Identifying 456 macular RPE-based DEGs in AMD

**Figure 1.**
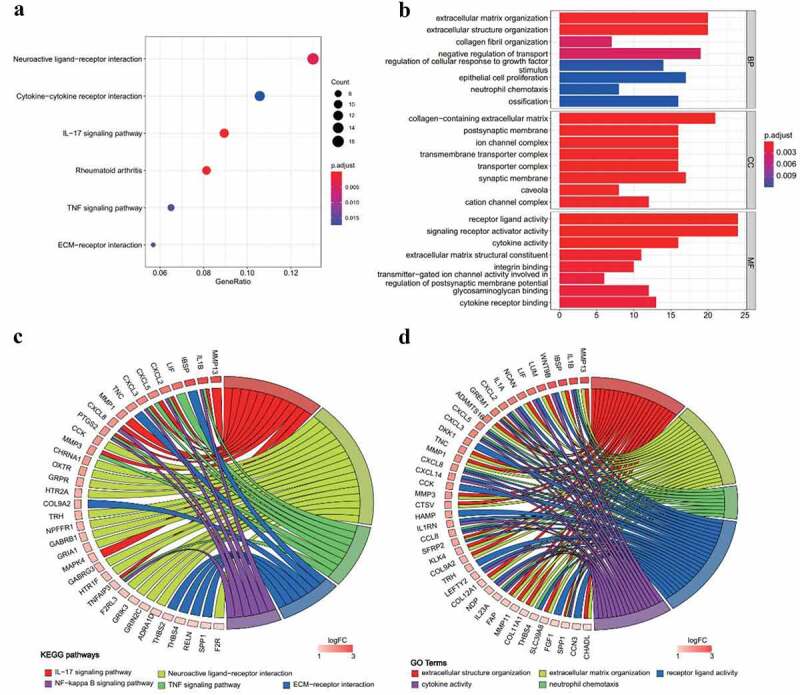
**GO and KEGG analysis results of upregulated genes in AMD**. (a) KEGG pathway enrichment results in AMD. Dotplot demonstrates enriched KEGG pathways of upregulated genes in AMD. (b) GO enrichment results in AMD. Barplot demonstrates upregulated enriched GO terms of biological process, cellular component and molecule function of upregulated genes in AMD. (c) Chord plot of representative KEGG pathways. (d) Chord plot of representative GO terms. The chords connect altered genes with enriched KEGG pathways or GO terms. The color of boxes next to gene labels indicates the log2 fold change. Adjusted p-value <0.05 was considered as significant

Macular RPE samples in RNA sequencing dataset (GSE135092) were selected (26 AMD, 105 normal) for identification of DEGs in RPE of AMD patients. A total of 456 significant DEGs were identified in which 201 genes were downregulated and 255 genes were upregulated (Supplement Figure S1, Supplement Table S2). Enrichment analysis was performed on DEGs. All enriched GO terms and KEGG pathways are presented in Table S3. KEGG analysis showed a significant enrichment of upregulated genes involved in interleukin-17 (IL-17) signaling pathway, neuroactive ligand-receptor interaction, tumor necrosis factor (TNF) signaling pathway and extracellular matrix (ECM)-receptor interaction (Figure 1a). The relationship between genes and representative KEGG pathways is presented in Figure 1c. GO analysis showed significant enrichment of extracellular structure organization, ECM organization, neutrophil chemotaxis, neutrophil migration, cytokine activity and chemokine activity (Figure 1b, Supplement Table S3). The relationship between genes and representative GO terms is shown in Figure 1d.

## M1 macrophages and activated memory CD4 T cells are positively correlated with inflammation

**Figure 2.**
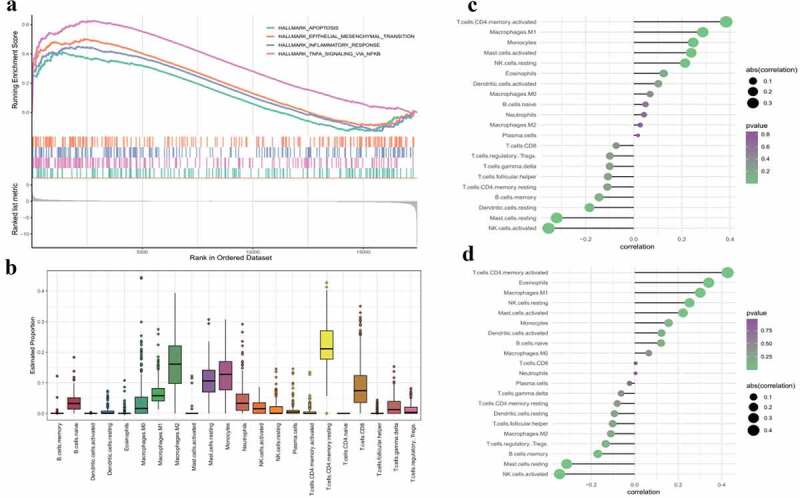
**GSEA results and immune cell infiltration in AMD**. (a)The GSEA results of representative gene sets associated with AMD. Adjusted p < 0.05 was regarded as significant. (b) Composition of immune cell types in RPE tissues. X-axis represent different immune cell types. Y-axis represent the estimated composition of immune cells. (c) Correlation analysis of GSVA scores of ‘inflammatory response’ gene set with composition of infiltrating immune cells. (d) Correlation analysis of GSVA scores of ‘TNF signaling via NF-κb’ gene set with composition of infiltrating immune cells. The size of dots is proportional to the absolute value of correlation coefficient. And the color of dots represents p value of correlation (greener indicates lower p value and purpler indicates higher p value). Correlation analysis was performed by Spearman correlation. A p-value <0.05 was considered statistically significant

GSEA was performed to explore biological differences between AMD and controls. Genes involved in apoptosis, epithelial-mesenchymal transition (EMT), inflammatory response and TNFα signaling through nuclear factor kappa B (NF-κb) were enriched in the AMD group (Figure 2a). In bulk RNA-seq, the expression level is a comprehensive result of all kinds of cells regardless of specific cell types. For both functional enrichment and GSEA results emphasis on inflammation in AMD, we used the CIBERSORT tool to explore the potential immune cell types involved in AMD. Estimation of abundances of 22 immune cell types is shown in Figure 2b. Compositions of activated mast cell and regulatory T cell were significantly different between AMD and controls (Supplement Table S4). Further, GSAV was performed to quantity enriched biological processes identified in GSEA (Supplement Table S5). GSVA scores of the ‘inflammatory response’ and ‘TNFα signaling through NF-κb biological processes’ were used to correlate with immune cell compositions. The findings showed that activated memory CD4 T cells and M1 macrophages were positively correlated with ‘inflammatory response’ (Figure 2c) and ‘TNFα signaling through NF-κb’ (Figure 2d). The positive correlation indicated that infiltration of CD4 T cells and M1 macrophage may be one of causes of inflammation in AMD.

## A total of 3718 RPE-based DMGs were identified in AMD

**Figure 3.**
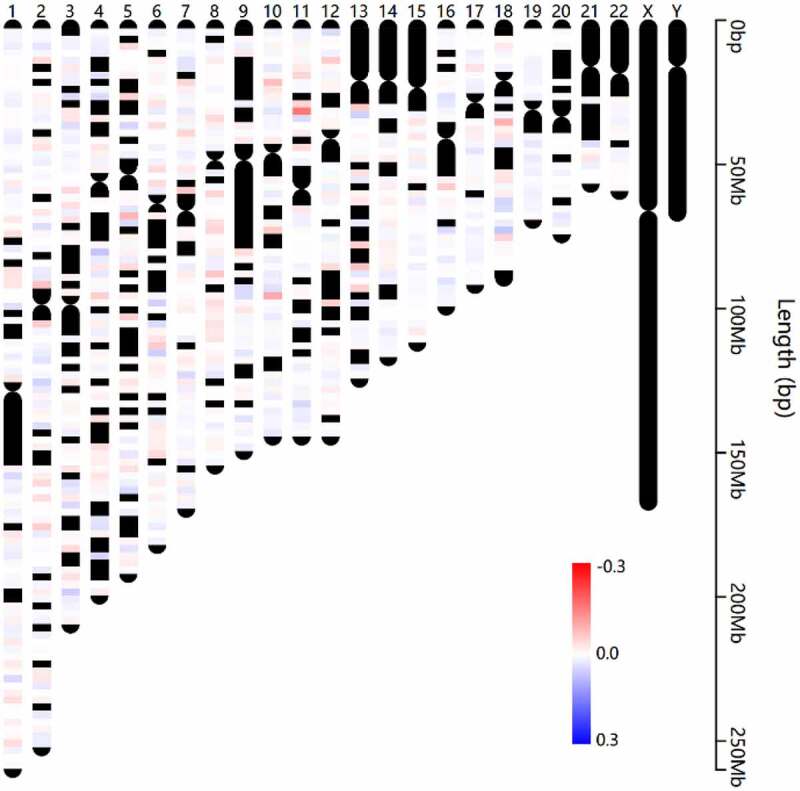
**Chromosome distribution of differentially methylated CpGs**. The color indicates the delta normalized beta value for AMD group comparing with control group. Red represents the hypermethylated sites in AMD, while blue represents the hypomethylated sites in AMD

**Figure 4.**
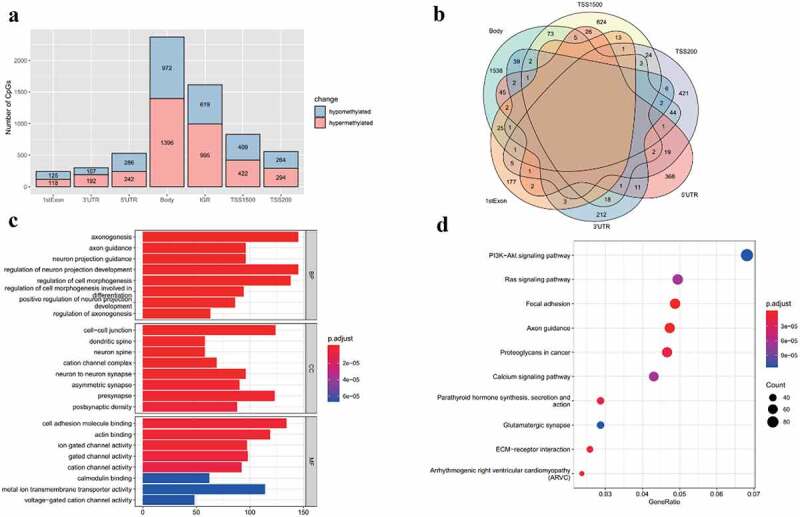
**Detailed locations of differentially methylated CpGs and enrichment analysis results of differentially methylated genes**. (a) Distribution of gene related regions of DMCs, including 1stExon, 3ʹUTR, 5ʹUTR, gene body, IGR, TSS1500 and TSS200. (b) Venn plot for gene related regions of DMCs. (c) Enriched GO terms of DMGs in AMD. (d) Enriched KEGG pathway of DMGs in AMD. Adjusted p-value <0.05 was considered as significant. UTR, untranslated region. IGR, intergenic region. TSS, transcriptional start sites

After rigorous filtering, normalized beta values of 412,481 probes were used for DNA methylation analysis. A total of 6441 CpGs met the differential methylation criteria in which 3659 CpGs were hypermethylated and 2782 CpGs were hypomethylated (Supplement Table S6). Chromosome distribution of these CpGs is shown in Figure 3. In addition, the regional distribution of these differential methylation CpGs was summarized. The findings showed that the preponderance of DMCs resides in the gene body region (Figure 4a). Notably, only intragenic CpGs (transcriptional start sites (TSS)1500, TSS200, 5ʹ untranslated region (UTR), 1stExon, body and 3ʹUTR) were further analyzed owing to the little knowledge of CpGs at the intergenic region (IGR) region. Therefore, 4827 DMCs were corresponding to 3718 DMGs. The findings showed that most DMCs were individually region-specific and DMCs at the gene body and TSS1500 shared the most common genes (Figure 4b).

GO and KEGG analyses were performed to further explore these DMGs (Supplement Table S7). The findings showed enriched GO terms such as axonogenesis, cell-cell junction and cell adhesion molecule binding (Figure 4c). In KEGG pathways, PI3K-Akt signaling pathway, Ras signaling pathway and ECM-receptor interaction were significantly associated with these DMGs (Figure 4d).

## Effect of DNA methylation on gene expression in AMD

**Figure 5.**
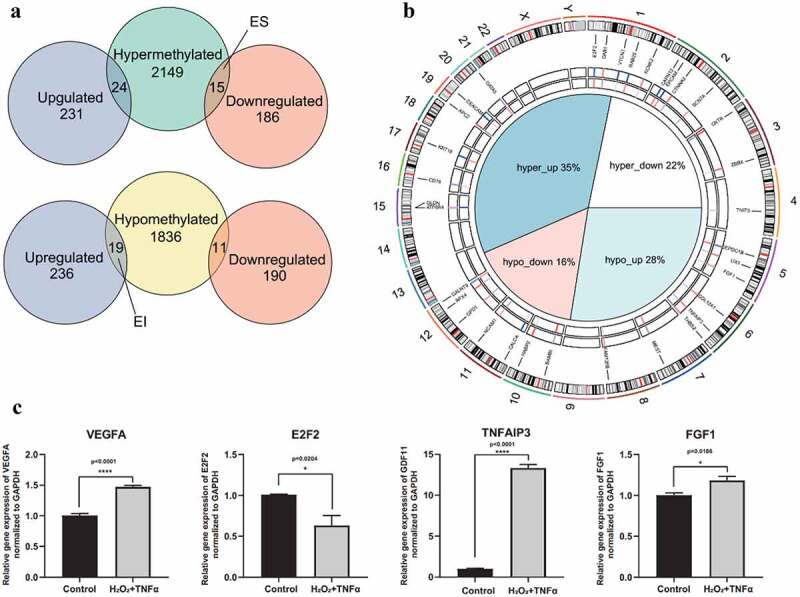
**Intersection of DEGs and DMGs**. (a) Intersection results of DEGs and DMGs. Venn plots depicting the intersection between hypermethylated genes and DEGs (top), hypomethylated genes and DEGs (bottom). 15 hypermethylated-downregulated genes as epigenetically suppressed (ES) genes and 19 hypomethylated-upregulated genes as epigenetically induced (EI) genes were identified. (b) Circular visualization of ES and EI genes. The central pie chart shows the proportion of overlap genes. The first tracks represent the delta of beta value of EI and ES genes. Red indicated hypermethylation in AMD, while blue indicated hypomethylation in AMD. The second track represents log2 fold change of EI and ES genes. Red indicates upregulated gene expression in AMD, while blue indicates downregulated gene expression in AMD. The third track is the gene symbols and corresponding chromosome position of EI and ES genes. The outer track is chromosomes. (c) Relative expression level of VEGFA, E2F2, TNFAIP3 and FGF1 under co-stimulation of H_2_O_2_ and TNFα. Barplots demonstrate that the expression level of VEGFA, E2F2, TNFAIP3 and FGF1 significantly altered under co-stimulation of H_2_O_2_ and TNFα (p < 0.0001, p = 0.0204, p < 0.0001, and p = 0.0186, respectively). Statistical difference between groups was assessed by Student’s t-test. p < 0.05 was considered statistically significant. (n = 4, mean ± SEM). *p < 0.05, **p < 0.01, ***p < 0.001, and ****p < 0.001 compare with control group

The first step of integrative analysis of DNA methylation and gene expression is intersecting DMGs and DEGs. Genes were classified based on the intersection results into four groups: hypermethylated-upregulated, hypermethylated-downregulated, hypomethylated-upregulated and hypomethylated-downregulated (Figure 5a). Generally, the methylation level is negatively correlated with gene expression level. The findings of the current study showed 15 hypermethylated-downregulated genes as ES genes (corresponding to 23 DMCs) and 19 hypomethylated-upregulated genes as EI genes (corresponding to 24 DMCs) (Supplement Table S8). Details on chromosome location, expression level and methylation level of the ES and EI genes are shown in Figure 5b.

To validate the expression level of these ES and EI genes, H_2_O_2_ and TNFα co-treated RPE were used to simulate oxidative stress and inflammation status *in vitro*, which are two critical factors in development and progression of AMD [36,37]. The level of VEGFA, a critical factor and therapeutic target of AMD was explored to determine reliability of the *in vitro* model. The findings showed that the level of VEGFA was significantly increased after co-treatment with H_2_O_2_ and TNFα (Figure 5c). Moreover, FGF1, E2F2 and TNFAIP3 genes were chosen to validate expression level of ES or EI genes by qPCR assay. The finding showed that the expression level of TNFAIP3 and FGF1 was significantly increased, while the expression level of E2F2 was significantly decreased under co-treatment with H_2_O_2_ and TNFα (Figure 5c). These results are consistent with the effect of DNA methylation on gene expression (Figure 5b).

## Two significant random forest classifiers were constructed

**Figure 6.**
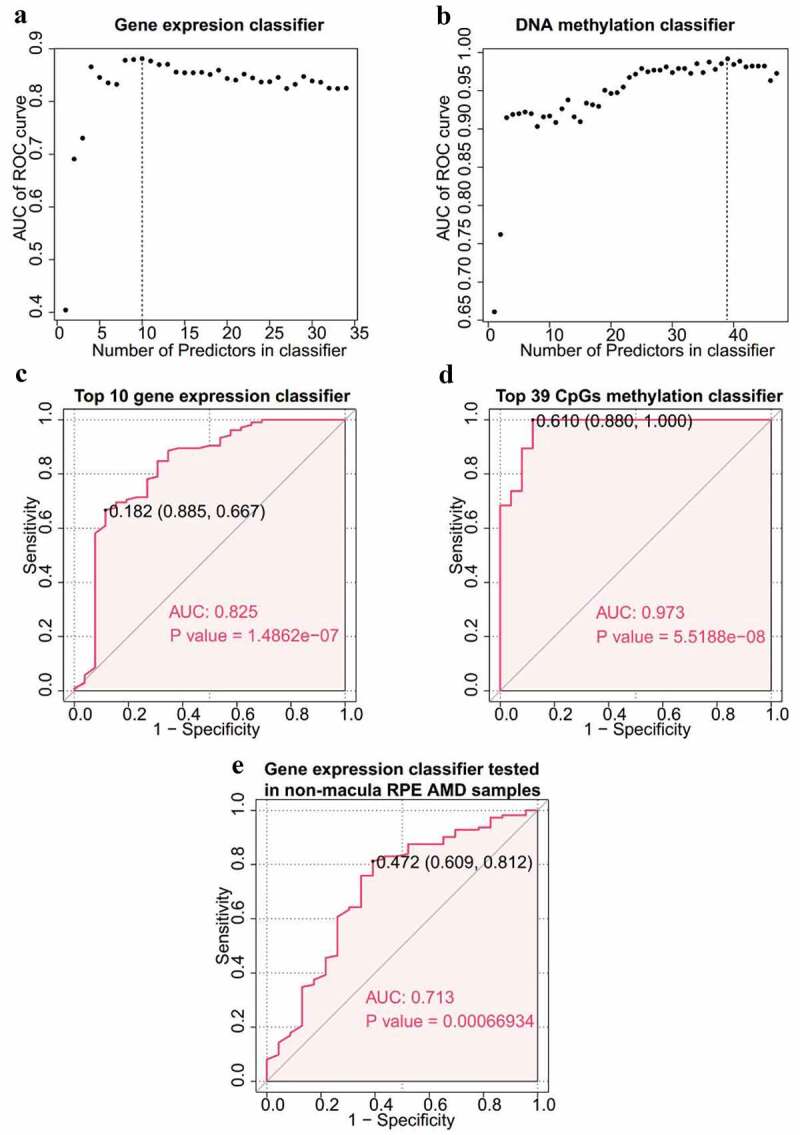
**Construction of random forest classifiers**. (a) Gene expression classifier. Scatter plot depicting the number of genes in gene expression classifier and corresponding predictive accuracy. The vertical dot line represents the top 10 genes with highest AUC of ROC curve. (b) DNA methylation classifier. Scatter plot depicting the number of CpGs in DNA methylation classifier and relative predictive accuracy. The vertical dot line represents the top 39 CpGs with highest AUC of ROC curve. (c) Top 10 genes based gene expression classifier. The AUC value is 0.825, and the p value is 1.4862e-07. The p value was calculated using the Wilcox-test method. (d) Top 39 CpGs based DNA methylation classifier. The AUC value is 0.973, and the p value is The AUC value is 0.825, and the p value is 5.5188e-08. (e) ROC curve of validation for gene expression classifier using non-macula RPE. The AUC value is 0.713 and the p value is 0.0007. ROC, receiver operating characteristic. AUC, area under the curve

Expression data of 34 EI and ES genes and 47 corresponding CpGs methylation data were used to construct two classifiers with random forest algorithm and LOOCV methods to distinguish AMD patients from normal controls. Importance of each variable was first determined and variables were ranked in descending order (Supplement Table S9). Variables were then added consecutively to identify the best predictors of classifiers by calculating the predicting power of each classifier. The top 10 genes had the highest predicting power of the gene expression classifier and the top 39 CpGs had the highest predicting power of the DNA methylation classifier (Figure 6a-6b). The ROC curve of the two classifiers with the best predictor are presented in Figure 6c-6d. Both classifiers showed significant classification capacity. The classifier based on DNA methylation data (AUC 0.973, *p-*value 5.5188e-08) showed higher predictive power compared with the gene expression classifier (AUC 0.825, *p*-value 1.4862e-07) (Figure 6c-6d). Gene expression data of 135 non-macular RPE samples from GSE135092 was then used as a test dataset to test robustness of the gene expression classifier. The ROC curve of the test dataset showed that the gene expression classifier was robust and was accurate using non-macular RPE (AUC 0.713, *p*-value 0.00066934, Figure 6e). This finding indicated that predictors from the EI and ES genes are potential RPE-sourced biomarkers for AMD.

## SMAD2 and NGFR were identified as hub gens through functional epigenetic module analysis

**Figure 7.**
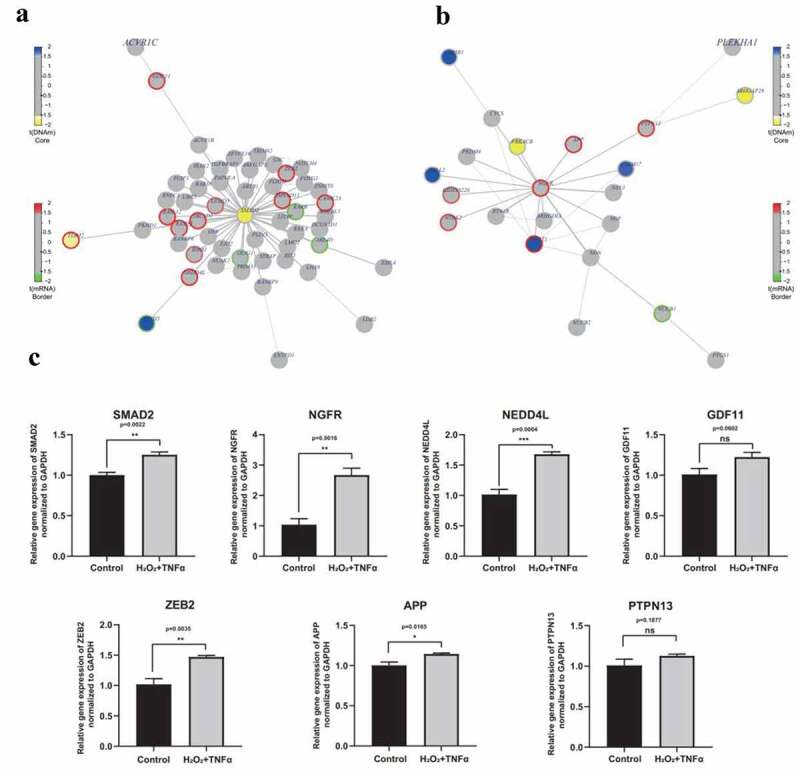
**Interaction networks and validation expression level of key genes**. (a) Network diagram of SMAD2 centric interaction network. (b) Network diagram of NGFR centric interaction network. Color of core of the node represents the t-statistics of the differential methylation analysis using FEM package. Blue indicates the hyper- hypermethylation in AMD group, while yellow indicates the hypo- hypermethylation in AMD group. Color of border of the node represents the t-statistics of the differential methylation analysis using FEM package. Red indicates the up-regulation of gene expression in AMD group, while green indicates the down-regulation of gene expression in AMD group. (c) Relative expression level of SMAD2, NGFR, NEDD4L, GDF11, ZEB2, APP and PTPN13 under co-stimulation of H_2_O_2_ and TNFα. Among these genes, the expression level of SAMD2, NGFR, NEDD4L, ZEB2 and APP significantly increased under co-stimulation of H_2_O_2_ and TNFα (p = 0.0022, 0.0016, 0.0004, 0.0035 and 0.0165, respectively). Although no significant difference is detected of GDF11 and PTPN13 (p = 0.0.0602, 0.1877, respectively), there is certain tendency for increased expression level in H_2_O_2_ and TNFα co-treatment group. Statistical difference between groups was assessed by Student’s t-test. p < 0.05 was considered statistically significant. (n = 4, mean ± SEM). *p < 0.05, **p < 0.01, ***p < 0.001, and ****p < 0.001 compare with control group

To further explore the role of DNA methylation and gene expression, FEM analysis was conducted to identify significant modules in the protein interaction network. Nine gene-centric interaction subnetworks were identified and the central genes were CD79B, EED, LCN12, MAPK12, NGFR, PEX19, SMAD2, SMURF2 and XYLT1 (Supplement Table S10). The main function and associated diseases of the nine central genes were explored and the results are summarized in Table 1. Although none of these genes was reported to be directly associated with AMD, NGFR and SMAD2 were selected for further analysis (Figure 7a-Figure 7b). SMAD2 mediates the signal of transforming growth factor (TGF)-β from cell membrane to nucleus [38]. Previous studies report that TGF-β pathway plays a significant role in occurrence and progression of AMD [39,40]. In addition, SMAD2 can be regulated by another central gene, SMURF2, which is a key member of the TGF-β signaling pathway [41]. NGFR, also known as p75NTR, is a receptor of neurotrophin which regulates various cell functions and is implicated in Alzheimer’s disease [42–44]. A link between Alzheimer’s disease and AMD has been previously reported [45–47]. This indicates that SMAD2 and NGFR are implicated in AMD. Further, expression level of SMAD2, NGFR and their interacting proteins in H2O2 and TNFα co-treated RPE was validated using qPCR. The findings showed that expression levels of NGFR and SMAD2 genes were significantly higher in H_2_O_2_ and TNFα co-treated RPE compared with controls (p = 0.0011, p = 0.001, respectively. Figure 7c). In addition, DNA methylation level of SMAD2 was hypomethylated in AMD group, whereas the gene expression was not different (Figure 7a). DNA methylation occurs prior to gene expression in epigenetic manner, thus the mRNA level of SMAD2 may increase with disease progression. Furthermore, expression of proteins integrated with SMAD2 and NGFR, such as NEDD4L, ZEB2, APP, GDF11 and PTPN13 were explore. Among these genes, the expression level of SAMD2, NGFR, NEDD4L, ZEB2 and APP increased significantly under co-stimulation of H_2_O_2_ and TNFα (p = 0.0022, 0.0016, 0.0004, 0.0035 and 0.0165, respectively) (Figure 7c). Although no significant difference is detected of GDF11 and PTPN13 (p = 0.0.0602, 0.1877, respectively), there is certain tendency for increased expression level in H_2_O_2_ and TNFα co-treatment group (Figure 7c). Expression levels of these integrating proteins was highly consistent with the findings from high-throughput data (Figure 7b). This finding indicated that SMAD2 and NGFR are potential markers in elucidating AMD pathogenesis. However, the detailed functions and mechanisms should be explored further.

**Table 1.** Characteristics of the nine candidate key genes

Gene	Full name	Main functions or pathways	Associated diseases
CD79B	CD79b molecule	B cell receptor signaling pathway	Lymphoma^[48,49]^
EED	Embryonic ectoderm development	Member of the epigenetic regulator gene family, transcriptional repression	Weaver syndrome, colorectal cancer^[50,51]^
LCN12	Lipocalin 12	Retinoic acid binding, long-chain fatty acid transport	Keratoconus^[52,53]^
MAPK12	Mitogen-activated protein kinase 12	MAPK signaling pathway	Cancer^[54,55]^
NGFR	Nerve growth factor receptor	Regulating apoptosis and activity of amyloid-beta	Alzheimer’s Disease^[43,44]^
PEX19	Peroxisomal biogenesis factor 19	Peroxisome biogenesis	Peroxisome biogenesis disorders^[56]^
SMAD2	SMAD family member 2	Transforming growth factor-beta signaling pathway	Colorectal cancer, cardiovascular disease^[57,58]^
SMURF2	SMAD specific E3 ubiquitin-protein ligase 2	SMAD-transforming growth factor-beta signaling	Cancer^[59,60]^
XYLT1	Xylosyltransferase 1	Biosynthesis of chondroitin sulfate and dermatan sulfate proteoglycans	Desbuquois dysplasia type II ^[61]^

## Discussion

AMD is a highly complex and disease resulting in vision loss, however, its pathogenesis has not been fully elucidated. Gene–environment interaction causes epigenetic modification and may play a significant role in AMD development [6]. Although several studies have reported novel methylation genes for AMD, a full combination with gene expression changes based on RPE tissues has not been explored [7–9,62]. Xu et al. [63]. identified several aberrant methylation-based biomarkers by integrating retina-based methylation data and gene expression data. RPE is the primary site of injury in AMD and promotes early development of the disease before retinal degeneration [64,65]. A recent study conducted ATAC-seq analysis and reported that global decreases in chromatin accessibility occur in the RPE in early stages of AMD and in the retina of advanced disease [66]. This implies that molecular alteration in RPE may be more sensitive and essential in understanding AMD pathogenesis. To the best of our knowledge, this is the first study to integrate DNA methylation data and transcriptome profiles based on RPE tissues to identify key genes in AMD pathogenesis.

The findings showed that upregulated genes in AMD were significantly enriched in inflammation-associated pathways, such as cytokine–cytokine receptor interaction, IL-17 signaling pathway and TNF signaling pathway. In addition, GSEA results showed inflammatory processes, including inflammatory response and TNFα signaling through NF-κb were significantly enriched. Studies report that immune activation, including innate immune responses, microglia activation and pathological parainflammation, significantly contribute to the AMD phenotype [67]. A previous review hypothesized that AMD is a consequence of the age-related random accumulation of molecular damage at the ocular level and subsequent systemic inflammatory host response [68]. GO analysis in the current study showed that upregulated genes were enriched in neutrophil chemotaxis, neutrophil migration and leukocyte chemotaxis. Previous studies have not explored blood-sourced inflammation mechanisms. Therefore, the current study explored immune cells infiltration of RPE in AMD patients. Gene sets of the inflammatory response and TNFα signaling through NF-κb were quantified using GSVA and then a correlation analysis was conducted between the genes and composition of immune cells. The correlation results showed that activated memory CD4 T cells and M1 macrophages were positively correlated with inflammatory response and TNFα signaling via NF-κb. A higher level of M1 macrophages chemokine transcript in advanced AMD macula has been reported previously [69]. M1 macrophage is a pro-inflammatory immune cell and is involved in secretion of cytokines, such as IL-6 and TNFα[70]. The findings of the current study showed that M1 macrophage is closely correlated with inflammatory response. A new kind of T-cell activation, ‘bystander activation’, was recently reported which is independent of the T-cell receptor (TCR) [71]. Several cytokines such as IL-1, IL-18 and IL-2 are potent activators of bystander activation. Notably, effector/memory CD4 T cells have a lower threshold of bystander activation compared with naïve CD4 T cells [72]. Bystander activation of CD4 T cells may play important roles in infection, autoimmunity and cancer [72]. However, the link between inflammation and infiltration of activated memory CD4 T cells in RPE of AMD has not been elucidated. Therefore, future AMD therapy should explore local inflammation and systemic inflammatory host response.

Moreover, GO analysis showed that extracellular structure organization and ECM organization are significantly associated with AMD. Basal deposits and accumulation of ECM between RPE and Bruch’s membrane (BrM) form in the early stages of the disease and are convert into drusen with progression of AMD [73]. RPE cells play a key role in secretion and remodeling of ECM components, and dysregulation of RPE cells triggers an inflammatory process by binding to active complement C3 [74]. The enrichment analysis results of DEGs indicated importance of inflammation and ECM in AMD, which was consistent with the findings of previous studies in the aspect of molecular changes of postmortem RPE samples.

Normalized beta values were used in differential methylated analysis to evaluate the methylation level. The number of hypermethylated CpGs was slightly higher compared with that of hypomethylated CpGs. In addition to the promoter region, a large number of DMCs are located in the gene body region. Concerning gene body methylation-associated regulation, all intragenic DMCs were selected for DMGs analysis, regardless of the specific region [23]. A total of 3718 DMGs were identified and selected for enrichment analysis. Enrichment analysis results showed that some enriched terms of DMGs were closely associated with AMD. There terms included PI3K-Akt signaling pathway and ECM-related terms, such as cell-cell junction, cell adhesion molecule binding and ECM-receptor interaction. This emphasizes the importance of ECM organization in AMD, which is consistent with DEGs enrichment analysis results. Although the detailed mechanism of PI3K-Akt pathway in AMD was not fully elucidated, it is reported that the PI3K-Akt signaling pathway mediated the formation of CNV and cell viability of RPE [75,76].

EI and ES genes were extracted by intersecting DEGs and DMGs. DNA methylation patterns are potential good indicators of early disease development and biological aging [77–80]. In the current study, two random forest classifiers were constructed to distinguish AMD from normal samples based on DNA methylation data and gene expression data. The two classifiers showed a significant predictive value. However, DNA methylation-based classifier was more sensitive compared with the gene expression-based classifier. The grade of AMD samples in DNA methylation were level 2 or 3 indicating that DNA methylation pattern is vulnerable and should be monitored in early stages of AMD. Environmental factors affect multiple tissues and epigenetic alterations may exhibit similar patterns in different tissues [6]. For example, methylation levels in PRSS50 promoter gene are similar in blood and retinal tissue in AMD patients [9]. This implies that the DNA methylation-based classifier constructed in the current study may work comparably in blood samples. Therefore, it may have a high clinical value in diagnosing AMD using blood samples. Notably, induced pluripotent stem cells (iPSCs)-derived RPE (iRPE) significantly revolutionized basic research and therapy of AMD [81,82]. Araki et al. [83]. compared the base-resolution methylome of iRPE and native RPE (nRPE) and reported that iRPE methylome closely resembled nRPE. Therefore, we speculate that the DNA methylation-based classifier could also be applied in evaluation of iRPE condition and for determining if patients qualify for clinical transplantation.

To further integrate DNA methylation and transcriptome profile, FEM analysis was performed and nine gene-centric subnetworks (CD79B, EED, LCN12, MAPK12, NGFR, PEX19, SMAD2, SMURF2 and XYLT1) were identified. Out of the nine ‘hotspots’, SMD2 and NGFR were identified as key genes (Table 1). SMAD2 is the downstream effector of the TGF-β signaling pathway. Wang et al. [84]. reported that TGF-β/SMAD2 signaling mediates formation of CNV through upregulation of VEGF and TNFα. EMT is a process induced by TGF-β signaling, which can lead to vascular fibrosis [39]. GSEA results showed that EMT was enriched in AMD patients. These processes are the main causes of vision loss in advanced stage of AMD. NGFR is involved in several cellular processes, such as apoptosis, cell survival and neurite outgrowth [85]. NGFR acts as an amyloid-beta (Aβ) receptor that mediates Aβ neurotoxicity and regulates Aβ production and deposition [86–88]. AMD is an age-related degeneration disorder thus it shares several common features with Alzheimer’s disease, including Aβ deposition, oxidative stress and inflammation [46,89]. In addition, expression level of SMAD2, NGFR and their integrating proteins was determined using H_2_O_2_ and TNFα co-treated RPE to simulate AMD-related oxidative stress and inflammation model *in vitro*. The findings showed that expression of NGFR and SMAD2 was significantly higher in H_2_O_2_ and TNFα co-treated RPE compared with controls. In addition, expression levels of integrating proteins were pretty consistent with levels shown through RNA-seq analysis using publicly available data. SMAD2 and NGFR may play a significant role in elucidating AMD pathogenesis and are potential therapeutic targets of AMD. To further explore the clinical significance of these two genes, studies should conduct biological assays and clinical validation, which are crucial for understanding pathogenesis of AMD.

Overall, A total of 456 DEGs and 4827 intragenic DMCs were identified in AMD. Enrichment analysis showed that upregulated genes in AMD were involved in IL-17 signaling pathway, TNF signaling pathway and ECM organization. Moreover, upregulated genes were enriched in systemic inflammatory response terms, such as neutrophil chemotaxis, neutrophil migration and leukocyte chemotaxis, which has not been reported in previous studies. The findings showed that immune infiltration was significant in AMD patients, and M1 macrophages, and activated memory CD4 T cells were positively correlated with inflammatory processes. In addition, DMGs were enriched in PI3K-Akt signaling pathway, cell-cell junction and ECM-receptor interaction. A total of 19 EI and 15 ES genes were obtained after intersection of DEGs and DMCs. Two significant random forest classifiers were constructed based on DNA methylation and transcriptome data. Analysis showed that the DNA methylation classifier seems was more accurate in distinguishing AMD from normal controls. To further integrate DNA methylation and transcriptome profile, FEM analysis was performed. SMAD2 and NGFR were selected as key genes. Furthermore, expression level of SMAD2, NGFR and their integrating proteins was validated in H_2_O_2_ and TNFα co-treated RPE. The findings showed that SMAD2 and NGFR are potential marker for pathogenesis and development of AMD therapy.

However, this study had limitations. First, this study did not explore all different stages of AMD, thus the characteristics of transcriptome and DNA methylation were not demonstrated with disease progress. Second, this study was based on two public datasets from two studies, thus biasness is inevitable in analytical strategies for integrating the different omics data. Third, the experimental validation conducted in the current study is preliminary. Further studies should be conducted to fully explore the roles of SMAD2 and NGFR in AMD.

## Conclusion

The current study integrated DNA methylation and gene expression data based on postmortem RPE samples. The findings showed that ECM organization, local inflammation and infiltration of specific immune cell types are implicated in pathogenesis of AMD. Furthermore, SMAD2 and NGFR are potential markers for exploring molecular mechanism and are potential therapeutic targets for development of AMD therapy.

## Supplementary Material

Supplemental Material

## Data Availability

All data generated during this study are included in this article and its supplementary files.
