# Supplementary material for: Integrated Analysis of DNA methylation and transcriptome profile to identify key features of age-related macular degeneration
Source: Bioengineered. 2021 Sep 27;12(1):7061–78. doi: 10.1080/21655979.2021.1976502 (PMC8806579; doi:10.1080/21655979.2021.1976502)
Supplement: Supplemental Material [file KBIE_A_1976502_SM4952.zip › supplementary/Supplement Table S1.docx]

Table 1. The primers for qPCR in this study.

|  | Forward(5’ to 3’) | Reverse(5’ to 3’) |
| --- | --- | --- |
| GAPDH | GTCTCCTCTGACTTCAACAGCG | ACCACCCTGTTGCTGTAGCCAA |
| VEGFA | TTGCCTTGCTGCTCTACCTCCA | GATGGCAGTAGCTGCGCTGATA |
| SMAD2 | GGGTTTTGAAGCCGTCTATCAGC | CCAACCACTGTAGAGGTCCATTC |
| NGFR | CCTCATCCCTGTCTATTGCTCC | GTTGGCTCCTTGCTTGTTCTGC |
| PTPN13 | GGATGAAGCCACTTACTCCAGC | CTCCAGGCTTAGGAGGTGATGA |
| APP | CCTTCTCGTTCCTGACAAGTGC | GGCAGCAACATGCCGTAGTCAT |
| NEDD4L | TAGCCTCAGCTCGCCAACAGTA | TAGCCTCAGCTCGCCAACAGTA |
| GDF11 | GCAAGTGCTACACAGCTGGTTC | CTCTAGGACTCGAAGCTCCATG |
| ZEB2 | AATGCACAGAGTGTGGCAAGGC | CTGCTGATGTGCGAACTGTAGG |
| FGF1 | ATGGCACAGTGGATGGGACAAG | TAAAAGCCCGTCGGTGTCCATG |
| E2F2 | CTCTCTGAGCTTCAAGCACCTG | CTTGACGGCAATCACTGTCTGC |
| TNFAIP3 | CTCAACTGGTGTCGAGAAGTCC | TTCCTTGAGCGTGCTGAACAGC |
