# Supplementary figures and images for: Integrated Analysis of DNA methylation and transcriptome profile to identify key features of age-related macular degeneration
Source: Bioengineered. 2021 Sep 27;12(1):7061–78. doi: 10.1080/21655979.2021.1976502 (PMC8806579; doi:10.1080/21655979.2021.1976502)

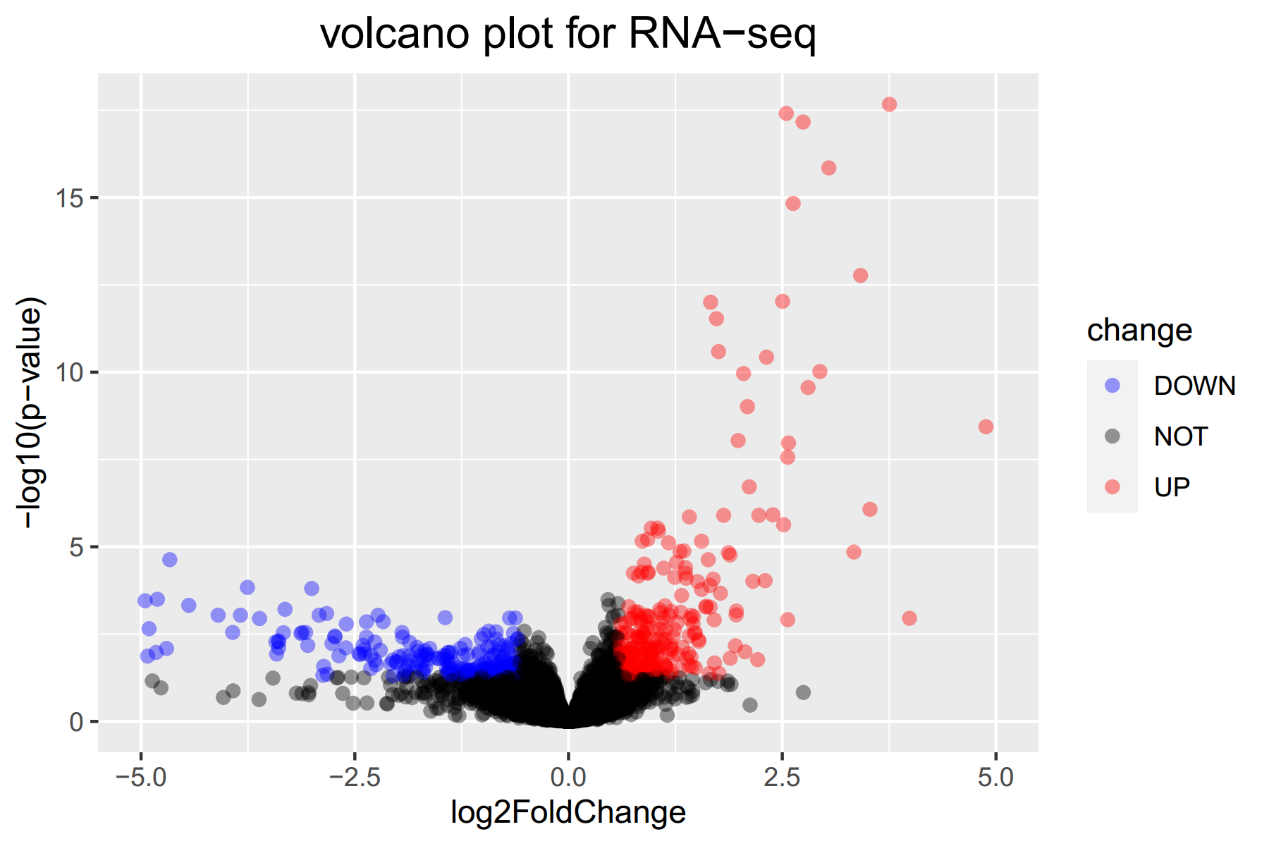

Supplement: Supplemental Material [file KBIE_A_1976502_SM4952.zip › supplementary/Figure S1.docx]
